# Multiple Coordinate Systems and Motor Strategies for Reaching Movements When Eye and Hand Are Dissociated in Depth and Direction

**DOI:** 10.3389/fnhum.2017.00323

**Published:** 2017-06-23

**Authors:** Annalisa Bosco, Valentina Piserchia, Patrizia Fattori

**Affiliations:** Department of Pharmacy and Biotechnology, University of BolognaBologna, Italy

**Keywords:** pointing movements, reference frames, visuomotor control, motor behavior, coordinate transformation, kinematics

## Abstract

Reaching behavior represents one of the basic aspects of human cognitive abilities important for the interaction with the environment. Reaching movements towards visual objects are controlled by mechanisms based on coordinate systems that transform the spatial information of target location into appropriate motor response. Although recent works have extensively studied the encoding of target position for reaching in three-dimensional space at behavioral level, the combined analysis of reach errors and movement variability has so far been investigated by few studies. Here we did so by testing 12 healthy participants in an experiment where reaching targets were presented at different depths and directions in foveal and peripheral viewing conditions. Each participant executed a memory-guided task in which he/she had to reach the memorized position of the target. A combination of vector and gradient analysis, novel for behavioral data, was applied to analyze patterns of reach errors for different combinations of eye/target positions. The results showed reach error patterns based on both eye- and space-centered coordinate systems: in depth more biased towards a space-centered representation and in direction mixed between space- and eye-centered representation. We calculated movement variability to describe different trajectory strategies adopted by participants while reaching to the different eye/target configurations tested. In direction, the distribution of variability between configurations that shared the same eye/target relative configuration was different, whereas in configurations that shared the same spatial position of targets, it was similar. In depth, the variability showed more similar distributions in both pairs of eye/target configurations tested. These results suggest that reaching movements executed in geometries that require hand and eye dissociations in direction and depth showed multiple coordinate systems and different trajectory strategies according to eye/target configurations and the two dimensions of space.

## Introduction

When we want to reach for an object in space, visual information about the object location is mapped within the early stages of the visual cortex in a coordinate system based on eye position (eye-centered coordinate system). Within parietal and premotor regions, information about the object location is transformed in extrinsic coordinates, taking into account hand and body position (hand and body-centered coordinate system). Thus, executing a movement towards a sensory target requires transformations between coordinate systems (Soechting and Flanders, 1992; Andersen et al., 1993; Andersen and Buneo, 2002).

Neurophysiological and behavioral investigations have targeted reaching movements to highlight principles underlying the process of coordinate transformations. In the neurophysiological field, many studies in monkeys have demonstrated the presence of parietal neurons encoding reaching actions in coordinate systems based on eye position (eye-centered; Snyder et al., 1997; Batista et al., 1999) as well as based on hand, body or target position (space-centered; Buneo et al., 2002, 2008; Marzocchi et al., 2008). Recently, a mixed coordinate system model, intermediate between eye- and space-centered coordinate systems, has been described in parietal areas of the monkey as representing a successful brain strategy that goes beyond the noise and variability generated by sensorimotor transformation (Deneve et al., 2001; Avillac et al., 2005; McGuire and Sabes, 2009, 2011; Mullette-Gillman et al., 2009; Chang and Snyder, 2010; Bosco et al., 2015a, 2016). This variety of coordinate systems used in parietal areas was found both for reaching targets located on a two-dimensional plane where targets varied the position only in direction dimension (Marzocchi et al., 2008; Bosco et al., 2015a) and also for reaching targets that varied the positions in depth (Hadjidimitrakis et al., 2014b; Bosco et al., 2016; Piserchia et al., 2017). Specifically, Bosco et al. (2016) demonstrated that a prevalent mixed encoding of target position exists within a population of neurons recorded in the posterior parietal area V6A of the macaque, using a task where reaching targets were decoupled from eye position in direction and depth.

In the behavioral field in humans, multiple coordinate systems (e.g., eye-, hand- and body-centered) were found for reaching towards both visual and proprioceptive targets (Beurze et al., 2006; Tramper and Medendorp, 2015; Mueller and Fiehler, 2016). Other human behavioral studies that investigated reaching movements executed to targets located in different depths and directions identified that the most suitable model able to maintain the spatial constancy was an eye-centered representation (Henriques et al., 1998; Medendorp and Crawford, 2002; Van Pelt and Medendorp, 2008).

All these behavioral works defined the coordinate system by measuring the reach error patterns for combination of eye, target, hand or body positions. However, the trajectories during the movement in configurations of eye and target positions that varied in direction and depth can be generated by common or different mechanisms adopted by the participants. Movement strategies were extensively investigated to define the role of sensory information on movement control and execution (e.g., Pelisson et al., 1986; Carlton, 1992), but little is known about the comparison of trajectory strategies for reaches that share the same eye/target relative positions or the same spatial position of the target. The trajectory strategies towards static targets can be defined as modification of trajectory paths across trial repetitions. This can be evaluated by the analysis of motor variability at various stages throughout the movement (i.e., peak acceleration (PA), peak velocity (PV) and peak deceleration (PD); Khan et al., 2002, 2003). The rationale of this method was that if reaching movements are programmed and not altered, movement variability should increase as the movement progresses (Khan et al., 2002, 2003). If corrections in the movement trajectory were made on the subsequent trial, the variability profiles would deviate from the programmed movement trajectory and differ between different stages of movement and across eye/target configurations.

Here, we tested different configurations of eye and target relative positions in depth and direction, using a task design that maximizes natural reaching conditions where objects are reached on a horizontal surface and at a comfortable distance. We explored whether different coordinate systems and trajectory strategies are employed to encode reach direction and depth. We compared reach errors patterns and trajectory variabilities for pairs of configurations that shared the same eye/target relative position and those that shared the same spatial target position. First, we identified multiple coordinate systems adopted to guide reaches directed at different directions and depths. Then, the comparison of variability distribution in pairs of eye/target configurations allowed us to quantify differences and similarities in the trajectory strategies across trials that were not evident from the simple comparison of the trajectory profiles.

## Materials and Methods

### Participants and Ethics Statement

Twelve right-handed participants (average laterality quotients: 0.90 [range 0.70–1.00]; Oldfield (1971)) with normal, or corrected to normal, vision (3 males and 9 females, age range: 23–42 years; mean age: 29.5 ± 6.92 years) completed this study. The participants had no history of musculoskeletal or neurological disorders. This study was carried out in accordance with the recommendations of the Bioethical Committee of the University of Bologna. All participants gave written informed consent in accordance with the Declaration of Helsinki.

### Apparatus and Stimuli

**Figure 1:**
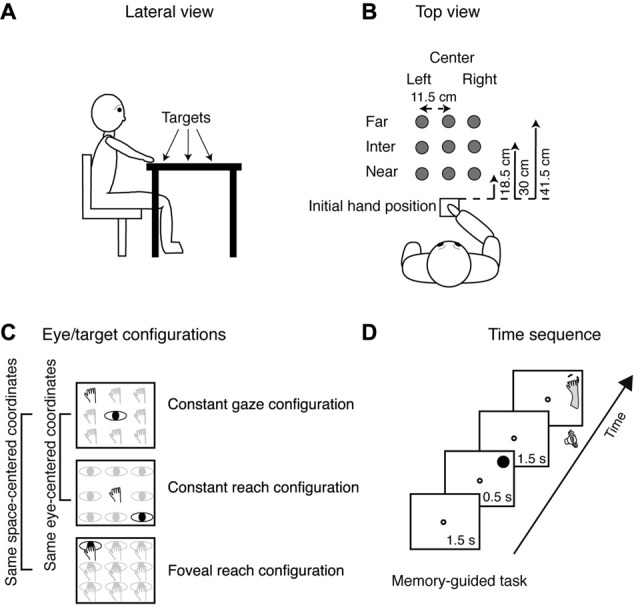
Experimental setup. **(A)** Lateral view of the target arrangement with respect to participant’s body. **(B)** Top view of the target arrangement in the peripersonal space. The participants performed reaching movements with their right hand towards one of the nine LEDs targets located at different depths (far, intermediate and near) and in different directions (left, center and right), gray dots. Reaching movements were performed in a dimly lighted room from the initial hand position located next to the body and marked by a squared perimeter, as indicated in the bottom. **(C)** Top view of the three task configurations. Top panel, Constant gaze configuration: reaching movements were performed towards one of the nine targets (hands). The spatial position of the target changed trial to trial, but gaze was kept constant at the central position. Central panel, Constant reach configuration: reaching movements were performed always toward the target located in the central position. During the execution of the task, participant had to fixate one of the nine different positions (eyes). Bottom panel, Foveal reach configuration: reaching movements were performed toward one of the nine targets. During the task, the participant had to fixate and reach the same target (eye and hand on the panel). The Constant gaze and the Constant reach configurations shared the same eye/target relative position (eye-centered representation); the Constant gaze and the Foveal reach shared the same spatial target position (space-centered representation). The two brackets on the left join parts of exemplary positions that share a common eye-centered coordinate system (first two panels) or a common space-centered coordinate system (first and last panels). **(D)** Time sequence of memory-guided reaching in the Constant gaze configuration. The small white dot represents the fixation target; the filled black dot represents the reaching target and the dashed dot represents the memorized location of the reaching target. The fixation target (a green LED) stayed on for 1.5 s and then the reaching target (a red LED) was turned on for 0.5 s in one of the nine locations. After 1.5 s from target offset, a sound indicated to the participant to reach with his/her right hand the memorized position of the target while maintaining fixation on the fixation target. The fixation target lasted until the participant completed the movement.

In all trials, the starting position of the hand (dominant right hand) was on a board placed adjacent to the touchscreen within a square marked with a tape and detectable by touch (size 12 × 12 cm) in front of the participant’s chest, as sketched in Figures 1A,B.

Reaching movements were performed in a dimly illuminated room. The head of the participants was supported on a chin rest in order to reduce movements. The stimuli were green (diameter 0, 3 cm) and red dots (diameter 1, 2 cm) presented at different depths and in different directions with respect to participant’s midline. The stimuli presented a luminance of ~17 cd/m^2^. Stimuli were presented on 19-inch touchscreen (ELO IntelliTouch 1939L) laid horizontally on a desk located at waist level with a visible display size of 37.5 × 30 cm and 15,500 touch points/cm^2^. The display had a resolution of 1152 × 864 pixels and a frame rate of 60 Hz.

Reaching movements were recorded using a motion tracking system (VICON motion capture system^®^) by sampling the position of two markers at a frequency of 100 Hz; markers were attached to the wrist (on the scaphoid bone) and on the nail of the index finger (reaching finger). The marker on the wrist was used to detect the onset and offset of reaching movements and was used to characterize the reaching component, as commonly done in kinematic studies on reaching (e.g., Roy et al., 2000; Gentilucci et al., 2001); the marker on the tip of the index finger was used for the kinematic analysis, as it was the hand portion that made contact with the target (e.g., Carey et al., 1996). Participants were asked to move at a fast but comfortable speed, and as accurately as possible.

Eye position was recorded using a Pan/Tilt optic eye-tracker (Eye-Track ASL-6000) recording real-time gaze position at 60 Hz. The participant’s dominant eye was illuminated by invisible infrared light, and the reflections were recorded by a video camera positioned 64 cm from the eye. The elevation distance between the eyes of participants and the touchscreen was 27 cm. During the task, fixation was additionally monitored on-line by the experimenter on all trials. Before collecting data from each participant, the equipment was calibrated using a nine-point grid. Participants were asked to fixate successively on each of a series of small dots arranged on three lines in the form of a square (23 × 23 cm).

### Behavioral Paradigm

Participants performed reaching movements on a desk where the touchscreen was positioned using their right hand. Figure 1A shows the target positions at different depths with respect to participant’s body. As depicted in Figure 1B, there were nine possible locations in which targets could appear: three placed at near distance (18.5 cm from the initial hand position) three at intermediate distance (30 cm from the initial hand position), and three at far distance (41.5 cm from the initial hand position). The targets were arranged in a square of 23 × 23 cm, and were located 11.5 cm apart each, either on the left or the right side, the central targets were placed along the sagittal midline. All targets were located within a comfortable reaching distance.

The task was composed of different eye/target configurations (see Figure 1C): the *Constant gaze configuration*, in which the eye fixated a central fixation target and the hand reached to one of the peripheral reaching targets (thus the reaching target was on the fovea only when presented at the same central location of the fixating target and in the periphery of the retina for the other eight possible locations); the *Constant reach configuration* in which the eyes fixated one of the peripheral targets and the hand always reached the central target; and *Foveal reach configuration* in which the fixation target and the reaching target were coincident. The Constant gaze and the Constant reach configurations were extrafoveal configurations in which the position of the fixation point was dissociated from the position of the reaching targets. These two configurations (eye-centered configurations) shared the same eye/target relative position and were used to study the eye-centered coordinate system (see the black exemplary positions in the first two panels in Figure 1C). The Foveal reach configuration instead was a configuration in which the position of the fixation point coincided with the position of the reaching target and shared the same spatial positions with the Constant gaze configuration. The pairs of targets in the same spatial position (space-centered configurations) allowed us to describe the space-centered coordinate system as it is indicated on the left of Figure 1C. On the left of Figure 1C the pairs of comparisons used to assess the two types of coordinate systems is indicated by grouping a pair of exemplary positions in the same eye-centered coordinates and a pair in the same space-centered coordinates. The combinations of the configurations as described above enabled us to define the coordinate systems as was done in neurophysiological experiments (Bosco et al., 2016).

We tested the participants in a memory guided reaching task as shown in Figure 1D. The sequence of the memory guided reaching consisted in the presentation of a fixation target (a green LED, diameter 0.3 cm) that stayed on for 1.5 s. Then, the reaching target (a red LED, diameter 1.2 cm) was flashed for 0.5 s. After 1.5 s from target offset, an acoustic signal indicated to the participant that they should reach the remembered position while maintaining fixation on the green-fixation target. The fixation point remained illuminated until the participant completed the movement.

The task was composed of five blocks of 27 trials. Within each block, trials of the three configurations were randomized. For stimuli presentation and data analysis, we used Matlab with the Psychophysics toolbox extension (Brainard, 1997).

### Data Analysis

After recordings, data positions were interpolated at 1000 Hz and were run through a fifth-order Butterworth low-pass filter (cutoff frequency, 30 Hz (Bosco et al., 2015b)). For data processing and analysis, we wrote custom software in Matlab (Mathworks) to compute the velocity profiles of all markers. Onset of movement was detected when wrist velocity remained above 5 mm/s for 200 ms; the following offset was detected when wrist velocity remained below 5 mm/s for 200 ms. Reach endpoints were extracted from touchscreen recordings; movement trajectory and variability from motion capture system recordings (Vicon^®^).

**Figure 2:**
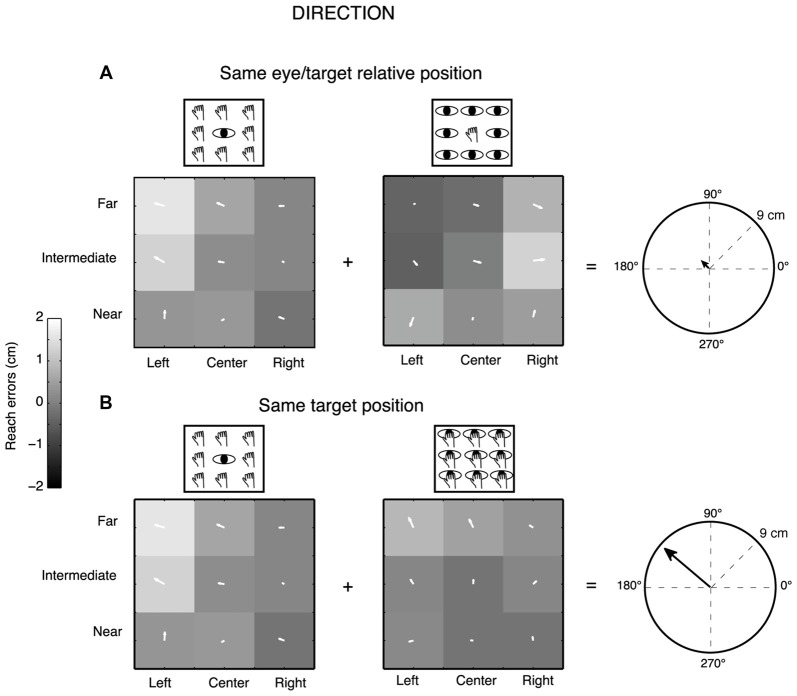
Gradient and vector analysis for real reach errors in direction in an exemplary participant. **(A)** The vector fields show the convergence for higher positive reach errors for targets to the left of the eye position in the Constant gaze and Constant reach mainly distributed along the direction dimension. The resultant vector on the right presents length of 0.91 cm. **(B)** The vector fields corresponding to Constant gaze and Foveal reach configurations show higher positive reach errors for left targets along the direction dimension. The resultant vector on the right presents length of 8.71 cm. See text for more details.

We measured two-dimensional reach endpoints and analyzed the horizontal (direction) and vertical (depth) dimensions of reach errors calculated by subtracting the respective horizontal and vertical coordinates of physical target location from the reach endpoint in that trial. We performed a gradient analysis (Peña and Konishi, 2001; Andersen and Buneo, 2002; Pesaran et al., 2006, 2010; Bremner and Andersen, 2014; Bosco et al., 2016) that has been applied previously to neural data to determine which combination of eye/target configurations (eye-centered and space-centered configurations) had the most influence on the pattern of reach errors or whether the configurations had equivalent influence. This technique allowed us to capture the complex geometry of task and extract the relevant features. 9/12 participants (7 females and 2 males, age range from 23 to 42 years) were included in this analysis, three participants were excluded because some target positions were discarded for missed detection of some endpoints by the touchscreen. In this case, the gradient analysis is not able to extract the values for the generation of the three vector fields corresponding to the three eye/target configurations for all the nine positions tested and so the analysis becomes less powerful. The gradient of reach error matrices (X-reach error matrices for direction dimension and Y-reach error matrices for depth dimension) was estimated with the Matlab gradient function and plotted as white arrows on the matrix elements. The directions and lengths of the set of white arrows indicate the relative importance of each variable on the reach error patterns of participants. Specifically, the direction of the arrows indicates whether reach errors in X or Y dimension were influenced by the near-far and/or the left-right target positions and the length of arrows is proportional to the magnitude of reach errors for each position. We performed separated gradient analysis for Constant gaze and Constant reach configurations to extract relevant spatial features for configurations that shared the same eye/target relative positions (eye-centered configuration) and for Constant gaze and Foveal reach configurations to extract spatial features for configurations that shared the same spatial target positions (space-centered configuration), as also simplified by the left part of Figure 1C. The x and y component of the two vector fields corresponding to the pair of the eye-centered and space-centered configuration, respectively, were summed together in order to obtain two resultant vectors (eye-centered and space-centered resultant vector) defined by the length. The two resultant vectors therefore indicate the overall contribution of the eye- and space-centered coordinate systems on the reach error pattern of each participant. For example, in Figure 2A, the Constant gaze and Constant reach matrices, the arrows reflect a reach error pattern that changes according to the eye position (eye-centered representation). As they point predominantly to left and right, respectively, they subtract one from the other so their sum is near zero (little arrow in the circle on the right of Figure 2A). In Figure 2B, the Constant gaze and Foveal reach matrices, the arrows indicate a reach error pattern that changes according to the spatial position of targets. As all the arrows point to the left, their sum is high (right of Figure 2B). This indicates an example of space-centered encoding of reaching for this participant.

The prevalent coordinate system employed by each participant was ascertained by randomization of the matrix elements (randomization test, 1000 iterations). The randomization allowed us to extract confidence intervals (CIs) that included the 99% of values for each pair of configurations analyzed (first pair: eye-centered configuration; second pair: space-centered configurations). This analysis is shown in Figure 4. As the eye-centered representation tends to have opposite directions of vector fields in Constant gaze and Constant reach configurations and consequently the resultant vector length is close to zero, we defined participants as encoding in eye-centered coordinates when the resultant vector was smaller than the lower CI for this pair of configurations (see Figure 4A). The space-centered representation assumes that the vector fields in Constant gaze and Foveal reach configurations have the same direction and consequently the vectors add together. We defined that there was a space-centered representation when the resultant vector was larger than the upper CI extracted from the sum of vector fields of Constant gaze and Foveal reach (see Figure 4B). The participants that showed resultant vectors not responding to previous criteria were defined as those encoding in mixed coordinate systems.

**Figure 3:**
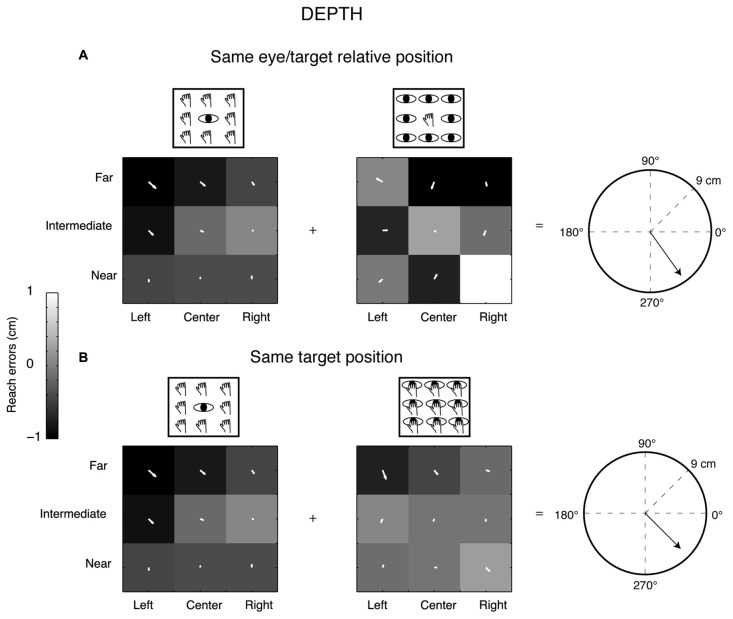
Gradient and vector analysis for real reach errors in depth. **(A)** The vector fields show higher positive reach errors for targets nearer than the fixation point in Constant gaze and not homogeneous distribution of reach errors in Constant reach configuration (arrows directed towards the bottom). The resultant vector on the right polar plot measures 7.93 cm. **(B)** Vector fields and resultant vector when reaching movements were made in the constant space-centered coordinates (the combination of Constant gaze and Foveal reach configurations). The pair of vector fields shows convergence for reach errors evoked by targets located near to the participant in both cases. The resultant vector length is 7 cm.

**Figure 4:**
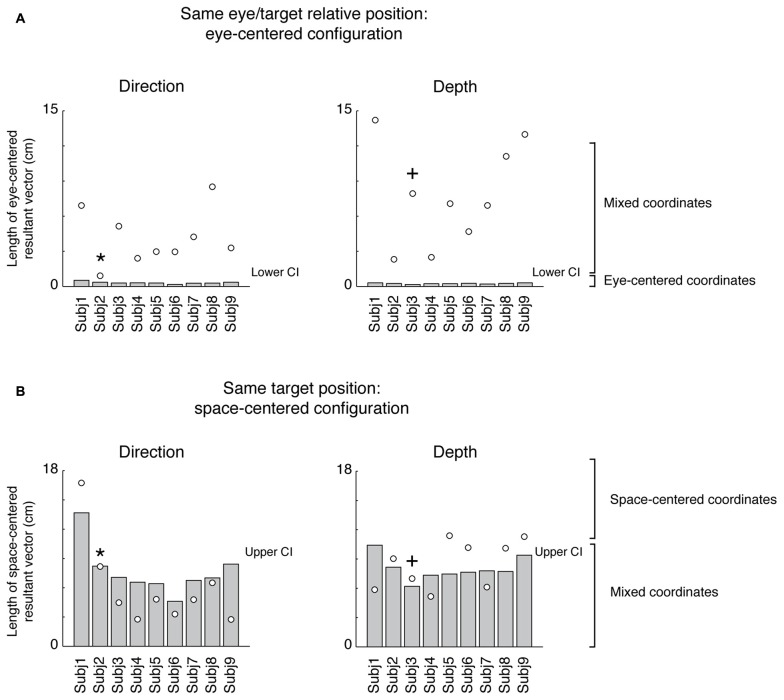
Vector length positions with respect to lower and upper confidence intervals (CIs). **(A)** Position of resultant vector lengths from the gradient and vector analysis extracted by the combination of Constant gaze and Constant reach configurations (eye-centered combination) of each participant (white dots) with respect to the lower CI (gray bar) for direction and depth, respectively. All values are above the lower CI. **(B)** Position of resultant vector lengths extracted by the combination of Constant gaze and Foveal reach configurations (space-centered combination) of each participant (white dots) with respect to the upper CI (gray bar) for direction and depth, respectively. Asterisks and crosses correspond to individual examples in Figures 2, 3, respectively. For direction, the majority of values fall below the upper CI; for depth, the majority falls outside the upper CI. For attribution criteria of eye-centered, space-centered and mixed coordinate systems see the text.

It is worthwhile noting that the term “space-centered” includes head-centered and world-centered coordinate systems, since the position of the target was kept constant with respect to the space, the head of the participant and the external world.

To assess the correspondence of results of the gradient analysis with more standard techniques to study reference frames at behavioral level, we performed a correlation analysis. The correlation analysis of reach errors has been previously applied in human and monkey behavioral studies (Scherberger et al., 2003; Beurze et al., 2006; Dessing et al., 2011). We reasoned that if the participants coded the movement in an eye-centered coordinate system, the reaching accuracy should be more similar in the configurations that share the same eye/target relative position compared to the configurations that share the same spatial target position. If the movement was encoded in space-centered coordinates, the reaching accuracy should be more similar in the configurations sharing the same spatial position of targets. We first calculated correlation coefficients for reach errors in depth and direction and then we extracted correlation coefficients for each participant, separately (Beurze et al., 2006; Dessing et al., 2011). This analysis estimates if the correlation coefficients are higher in eye-centered or space-centered coordinate systems in each participant when pointing to targets placed at different locations relative to the body and with gaze fixed at different directions. Specifically, we compared the similarity of reach errors among configurations presenting the same eye/target relative position (Constant gaze and Constant reach configuration) and configurations presenting the same spatial positions of targets (Constant gaze and Foveal reach configuration) as described for gradient analysis. To evaluate whether correlation coefficients were more deviated towards an eye-centered or a space-centered coordinate system with respect to diagonal, we calculated the average correlation coefficients across participants in depth and direction separately, and corresponding CIs were estimated by the standard deviations of the data.

As measure of movement corrections along the motor execution, we performed an analysis of the variability of trajectories across trials. For each participant, (*N* = 12) we calculated standard deviations across trials in both Y (depth) and X (direction) dimensions at four relevant points of movement for trajectories described by the marker located on the index finger and that corresponded to: peak acceleration (point of maximum acceleration, PA), peak velocity (point of maximum velocity, PV), peak deceleration (point of maximum negative acceleration, PD) and the end of movement (END); then we averaged across the participants (Khan et al., 2002, 2003; Kasuga et al., 2015). We compared the distribution of spatial variability for eye-centered configurations (Constant gaze and Constant reach configurations) and for space-centered configurations (Constant gaze and Foveal reach configurations) in depth and direction by a two-way ANOVA (2 eye/target configurations × 4 points on the trajectory) and by a Bonferroni *post hoc* test when the interaction was significant.

We carried out the three-way ANOVA on the trajectory variabilities separated for eye-centered and space-centered configurations with reach dimension as factor 1 (2 levels, depth and direction), eye/target configuration as factor 2 (2 levels, Constant gaze and Constant reach in eye-centered configuration and Constant gaze and Constant reach in eye-centered configuration, respectively) and points on the trajectory as factor 3 (4 levels, PA, PV, PD and END) to assess the interaction of these three factors on movement variability.

We included the central positions of the two constant configurations only in the qualitative data of gradient analysis (Figures 2, 3). We excluded them from the calculation of resultant vectors and trajectory variabilities.

## Results

We used a three-dimensional memory-guided reaching task with nine target locations that participants had to reach with different combinations of eye and target position, for a total of 27 types of trials. Reaching targets were located on a table arranged at a comfortable distance from the participants’ body that allowed a natural interaction with targets, as shown in Figure 1.

### Analysis of Reach Error Patterns

To define the predominant coordinate system employed by each participant and characterize the pattern of reach errors in depth and direction dimension, we used a combination of gradient and vector analysis which has been used by other authors to describe the influence of more than one variable simultaneously (Pesaran et al., 2006, 2010; Bremner and Andersen, 2014; Bosco et al., 2016). Figure 2 shows example vector correlations derived from one exemplar participant. Three 3 × 3 gradient fields represent the reach errors along the X dimension (direction) for every configuration of eye and reaching target position in each of the three tasks tested. Each element within the matrices therefore represents the gradient plotted as two-dimensional vector fields. We calculated the length of resultant vector as the sum of the x and y component of each arrow forming the vector field pair (Constant gaze/Constant reach configurations; Constant gaze/Foveal reach configurations). The fields in Figure 2A depict the two configurations sharing the same eye/target relative position. The two vector fields show opposite direction mainly distributed on the X dimension. In particular, the participant exhibits higher positive X-reach errors for targets located to the left of eye position in both configurations. This suggests that the reach error pattern changed according to the eye position (eye-centered coordinates). The sum of the vector fields tends to give a small resultant vector in length (Figure 2A, polar plot on the right; eye-centered resultant vector = 0.91 cm) since the two fields are characterized by arrows pointing into opposite direction and subtract each other. In Figure 2B, the combination of Constant gaze and Foveal reach, that shared the same spatial position, showed a similar alignment of vector fields that pointed towards left targets. In this specific case, the alignment of vector fields suggests a reach error pattern based on the spatial position of the target not dependent on the eye (space-centered coordinates). For this combination, the sum of vector fields generates a larger resultant vector because the vector fields are characterized by similar directions of the arrows and add together (space-centered resultant vector = 8.71 cm, Figure 2B, polar plot on the right). This example suggests the presence of two coordinate systems according to the combination of eye/target configurations.

Figure 3 shows the same type of analysis but for a different participant and in contrast to the previous example we considered the depth dimension (Y-reach errors) rather than the direction. This participant showed a different pattern of vector fields of reach errors when we considered the Y reach errors (depth). In this case, the vector field combination of the configurations sharing the same eye/target relative position (Constant gaze and Constant reach configuration, Figure 3A) did not show a specific alignment suggesting a reach error distribution not consistent with the eye-centered pattern (eye-centered resultant vector = 7.93 cm). The vector fields in the same target configuration (Constant gaze and Foveal reach, Figure 3B) showed the same alignment with arrows that mainly pointed towards the near positions with the space-centered resultant vector equal to 7 cm (Figure 3B, polar plot on the right). In this case, the analysis of vector fields suggests a reach error pattern more based on the target position (space-centered coordinates) rather than on the eye/target relative position (eye-centered coordinates).

In order to statistically determine the prevalent coordinate system employed by each participant, we resampled the resultant vector lengths to obtain lower and upper CIs that included 99% of values for each pair of configurations analyzed (Figure 4). The eye-centered resultant vector was derived from the sum of vector fields of Constant gaze and Constant reach configurations (Figure 4A), whereas the space-centered resultant vector resulted from the sum of vector fields of Constant gaze and Foveal reach configurations (Figure 4B). The eye-centered representation tends to have opposite directions of vector fields corresponding to reach errors varying according to the eye position. We defined the eye-centered coordinate system when the resultant vector of Constant gaze and Constant reach combination was shorter than the lower CI for this pair of configurations. We then defined the space-centered coordinate system when the resultant vector of Constant gaze and Foveal reach combination was larger than the upper CI. In this way, the lower and the upper CIs were extracted from two different distributions corresponding to the two combinations used. The positions of eye-centered resultant vectors (Constant gaze + Constant reach configurations), for direction and depth dimensions, are represented in Figure 4A as white dots. In our hypothesis, significant eye-centered representation included eye-centered resultant vector smaller than the lower CI. From Figure 4A it is evident that this never happened in our study as all participants showed eye-centered resultant vectors larger than the lower CI in both direction and depth. Figure 4B shows the positions of space-centered resultant vectors (white dots) with respect to the upper CI. In direction, one out of nine participants presented the resultant vector larger than the upper CI and six out of nine participants in depth; so 11% of participants used space-centered coordinates to encode the direction of reaching target and 89% used mixed coordinates. We found that the majority of tested participants (67% of participants) encoded the depth of target position using space-centered coordinates and 33% of participants using mixed system coordinates.

An interesting aspect is represented by the number of participants that changed or maintained the same type of coordinate system across depth and direction dimension. The majority of participants (77%) used different coordinate system in depth and direction and 23% used the same coordinate system. Among participants that changed the coordinate system, 14% of participants switched from space-centered representation in direction to mixed in depth; 86% of the participants switched from the mixed coordinates in direction to space-centered coordinates in depth.

**Figure 5:**
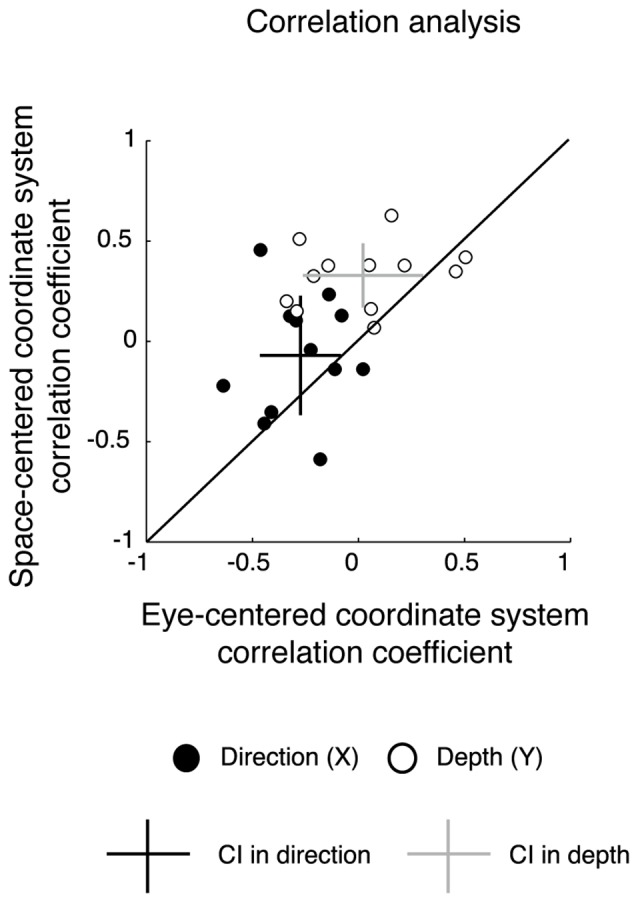
Correlation analysis. Correlation coefficients for eye-centered coordinates vs. space-centered coordinates for all participants. Each data point represents the value of the correlation coefficient of each participant in direction (black) and depth (white). Black cross represents the average correlation coefficient in direction with the corresponding CIs; gray cross represents the average correlation coefficient in depth with the corresponding CIs. For depth the CIs do not cross the diagonal, indicating a significant representation in space-centered reference frame, whereas for direction, intervals cross the diagonal, indicating a mixed representation.

The vector fields in the study of coordinate systems adopted by the brain have been mostly used for the analysis of neural data and, to our knowledge, have never been applied to behavioral variables (e.g., reach errors). However, to assess the reliability of the vector field analysis in a way more standardized for behavioral studies, we calculated correlation coefficients on reach errors in order to compare the similarity of reach errors among configurations presenting the same eye/target relative position (Constant gaze and Constant reach configurations) and configurations presenting the same spatial positions of the targets (Constant gaze and Foveal reach configuration). In Figure 5, we plotted the correlation coefficients for each participant in eye-centered vs. space-centered coordinate systems for reach errors in depth (white circles) and direction (black circles). The majority of points were located on the upper side of diagonal suggesting higher correlation for reach errors in space-centered coordinates. Additionally, we calculated the averaged correlation coefficient with the corresponding CIs (represented as crosses in Figure 5). In depth, the correlation coefficient averaged across participants and relative to the eye-centered coordinate system was 0.02 ± 0.28 (*P* < 0.05 in 5/12 participants). For the space-centered coordinate system, the averaged correlation coefficient was 0.33 ± 0.16 (*P* < 0.05 in 3/12 participants). In direction, the averaged correlation coefficients were −0.27 ± 0.16 for the eye-centered coordinate system (*P* < 0.05 in 3/12 participants) and −0.07 ± 0.29 for the space-centered coordinate system (*P* < 0.05 in 3/12 participants), respectively. In general, CIs of averaged correlation coefficients in depth did not touch the diagonal indicating a preponderance of the space-centered representation while, in direction, the CIs crossed the diagonal indicating a mixed representation.

The results from the correlation analysis confirm the previous results of vector analysis by showing a preponderant space-centered representation for depth and a mixed representation for direction.

### Analysis of Movement Variability

**Figure 6:**
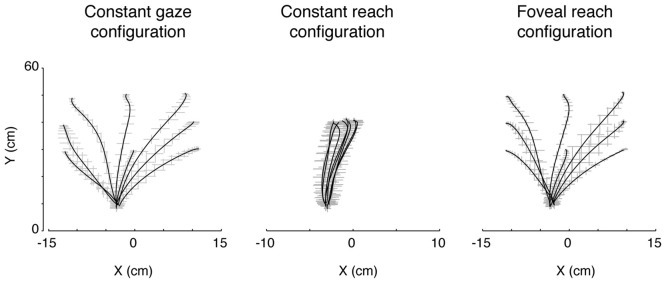
Actual trajectories of the movement. The trajectories of the index finger during movement in the three eye/target configurations in one participant. Solid lines indicate the averaged trajectory and gray crosses the X and Y variabilities along the movement. Note that, although the square of the initial hand position was on the midsagittal line (see Figure 1B), as all participants held the hand horizontally rotated with respect the midline of the screen, the marker of the index resulted more deviated to the left with respect to the origin of the axes defined, by system calibration, on the center of initial hand position square.

**Figure 7:**
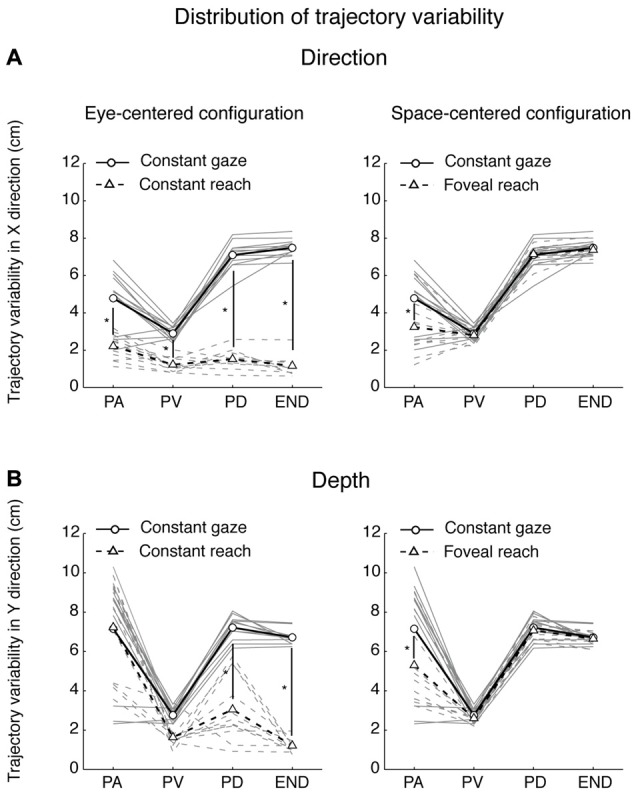
Spatial variability analysis. **(A)** Distribution of variability (cm) in the X dimension (Direction) for pairs of movements that were identical in eye-centered coordinates (left) and of pairs of movements towards targets having the same position in space-centered coordinates (right). Circles connected by solid lines correspond, on the left and on the right, to Constant gaze averaged variabilities. Triangles connected by dotted lines correspond, on the left, to Constant reach averaged variabilities and, on the right, to Foveal reach averaged variabilities. Gray solid and dotted lines indicate, on the left, individual variabilities of each participant for Constant gaze and Constant reach configurations, respectively and, on the right, Constant gaze and Foveal reach configurations, respectively. PA, peak acceleration; PV, peak velocity; PD, peak deceleration; END, end of movement. **(B)** Distribution of variability (cm) in the Y dimension (Depth) for pairs of movements that were identical in eye-centered coordinates (left) and of pairs of movements towards targets having the same position in space-centered coordinates (right) as in **(A)**. All conventions are as in **(A)**. Asterisks indicate significant Bonferroni *Post hoc* test when two-way ANOVA interaction was significant, *P* < 0.05.

Participants performed smooth trajectories to acquire target positions in each of the three different eye/target configurations, as reported in the individual example of Figure 6. Our hypothesis was that the distribution of variability, as a measure of modifications of trajectory paths across trials, could be different across the three eye/target configurations and space dimensions (direction and depth). For this reason, we analyzed the variability in the trajectories of each participant (*N* = 12) at relevant points during the movement (PA; PV; PD) and at the end of the movement (END; Khan et al., 2002, 2003). Figure 7 shows the average of variability distributions in black and individual participant variability distribution in gray. We then performed a two-way ANOVA on trajectory variabilities separately for pair of configurations sharing the same eye/target relative position (eye-centered configurations; Figure 7, left) and for the two that shared the same spatial target position (space-centered configuration; Figure 7, right) and for direction and depth (Figures 7A,B, respectively). This statistical analysis allowed to assess whether the distributions of variabilities differed between the pair of configurations sharing the same coordinate system. In direction, we carried out the two-way ANOVA on the trajectory variabilities with the eye-centered configuration as factor 1 (Constant gaze and Constant reach, 2 levels) and the points on the trajectory as factor 2 (PA, PV, PD and END, 4 levels). We found main effects of the eye-centered configuration (*F*_(1,3)_ = 716.53, *P* < 0.05) and of the points on the trajectory (*F*_(1,3)_ = 49.80, *P* < 0.05). In addition, we found significant two-way interactions between eye-centered configurations and points on the trajectory (*F*_(1,3)_ = 56.25, *P* < 0.05). The distribution of variabilities in Constant gaze and Constant reach (Figure 7A, left) significantly differed in all the four points on the trajectory (Bonferroni *post hoc* test, *P* < 0.006). For direction, we compared the trajectory variabilities by the two-way ANOVA with the space-centered configuration as factor 1 (Constant gaze and Foveal reach, 2 levels) and the points on the trajectory as factor 2 (PA, PV, PD and END, 4 levels). We found main effects of space-centered configuration (*F*_(1,3)_ = 6.04, *P* < 0.05) and of the points on the trajectory (*F*_(1,3)_ = 173.02, *P* < 0.05). The analysis showed a significant interaction between space-centered configurations and points on the trajectory (*F*_(1,3)_= 7.15, *P* < 0.05). The multiple comparison analysis revealed a significant difference only at the PA between Constant gaze and Foveal reach configurations (Figure 7A, right, Bonferroni *post hoc test*, *P* < 0.006).

In depth, for eye-centered configuration, the two-way ANOVA analysis showed significant main effects of eye-centered configuration (Constant gaze vs. Constant reach; *F*_(1,3)_ = 77.35, *P* < 0.05) and of points on the trajectory (PA, PV, PD and END; *F*_(1,3)_ = 47.17, *P* < 0.05) and significant interaction was found between these two factors (*F*_(1,3)_ = 18.09, *P* < 0.05). The multiple comparison analysis revealed that the variabilities at the PD and at the END were significantly different between Constant gaze and Constant reach configuration (Figure 7B, left, Bonferroni *post hoc* test, *P* < 0.006). When we considered the space-centered configuration, we found significant main effects of space-centered configuration (Constant gaze vs. Foveal reach; *F*_(1,3)_ = 4.60, *P* < 0.05) and of points on the trajectory (PA, PV, PD and END; *F*_(1,3)_ = 61.38, *P* < 0.05). A significant interaction was found between the two factors (*F*_(1,3)_ = 2.77, *P* < 0.05) and the multiple comparison analysis showed significant difference in variabilities only at the PA between Constant gaze and Foveal reach configurations (Figure 7B, right, Bonferroni *post hoc* test, *P* < 0.006).

The distributions in Figures 7A,B illustrate how different or similar the movement variabilities were within each pair of eye/target configurations in direction (Figure 7A) and depth (Figure 7B). In direction, the movement variability showed that in space-centered coordinates the distribution of variabilities was similar from the peak velocity to the END (Figure 7A, right). The variability in eye-centered coordinates was completely different for the entire duration of the movement, meaning that the participants used two different strategies in approaching the targets (Figure 7A, left). On the contrary, in depth, the distribution of variabilities in eye-centered coordinates was similar for the first part of the movement (Figure 7B, left) and in space coordinates overlapped from the peak velocity to the END (Figure 7B, right). All comparisons show a consistent variability distribution across participants (Figure 7, gray lines).

We then performed the three-way ANOVA to assess whether the trajectory variabilities were influenced by the interaction between the dimensions of reach (direction and depth), the eye/target configurations and the points on the trajectory (see “Materials and Methods” Section). In eye-centered configuration, we found significant interactions between dimensions of reach (direction and depth) and eye-centered configurations (*F*_(1,3)_ = 15.82, *P* < 0.05) as well as between dimensions of reach and points on the trajectory (*F*_(1,3)_ = 28.33, *P* < 0.05). In space-centered configuration, we found significant interaction between dimensions of reach and points on the trajectory (*F*_(1,3)_ = 17.32, *P* < 0.05). All these results suggest that movements along direction and depth dimensions depend on different trajectory strategies in eye- and space-centered coordinates. In general, the motor strategies across trials adopted by each participant were influenced by the eye/target relative configurations and by the reach dimension (direction and depth).

## Discussion

In the present study, we examined different aspects of the encoding of memory-guided reaching movements to targets placed at different depths and different directions. The specific set of experimental configurations, dissociating or consistent, in eye and target positions, allowed us to analyze the predominant coordinate system used in a reaching setup very similar to natural conditions that maximized an easy interaction with the targets.

Our results reveal a predominant use of mixed coordinate system when different directions are considered, whereas space-centered coordinate system predominates for changes in depth. To assess this, we applied a combination of vector and gradient analysis that is typically used for neural data (Pesaran et al., 2006; Bremner and Andersen, 2014; Bosco et al., 2016) but, to our knowledge, it is the first time that these analyses have been used for behavioral data. The analysis of the combination of vector fields across the different eye/target configurations (Figures 2–4) highlighted that both eye-centered and space-centered representations were present, but space-centered was predominant in depth and the two coordinate systems showed the same influence in direction (mixed coordinate system, see Figure 2 for an example). Our laboratory (Bosco et al., 2016) investigated the encoding of reaching target located at different depths and directions in a parietal area of the macaque and we found both eye- and space-centered representations differently balanced across neurons, similar to present behavioral results.

As mixed coordinate systems have been found in the complex network of parietal regions (Stricanne et al., 1996; Buneo et al., 2002; Cohen and Andersen, 2002; Battaglia-Mayer et al., 2003; Avillac et al., 2005; Mullette-Gillman et al., 2005, 2009; Chang and Snyder, 2010; McGuire and Sabes, 2011; Hadjidimitrakis et al., 2014b; Bosco et al., 2015a, 2016; Piserchia et al., 2017) and this parallels the present behavioral data, it can be suggested that what we have found here is the outcome, at behavioral level, of the neural discharges investigated in those works. The impact of mixed representations show several advantages, as described in several modeling and human studies. In fact, some authors described that, if the system can simultaneously represent different coordinate systems, the noise associated with coordinate conversion is strongly reduced (Deneve et al., 2001; Avillac et al., 2005; McGuire and Sabes, 2009, 2011; Bernier and Grafton, 2010; Beurze et al., 2010; Blohm, 2012; Buchholz et al., 2013). In addition to noise reduction, a further advantage of flexible coordinate systems derives from evidence that the computation of the motor response is complicated due to the necessity to integrate signals in retinal, proprioceptive, and motor coordinate systems (Buneo et al., 2002) and the brain requires some time to converge on a correct movement vector calculation. The adoption of a coordinate system that simultaneously takes into account different landmarks allows successfully fast movement corrections. Here, the prevalent use of mixed coordinate systems in direction is interesting because we have compared two configurations that shared the same eye/target relative position but that required different organization of movement trajectories: in one configuration participants reached the peripheral positions and in the other always the central position. This suggests that, in direction, the relative position of eye and target strongly contributes to spatial coordinates rather than the dynamical requirements of movement trajectories. Overall, our results support the view that spatial locations are represented in multiple coordinate systems with their individual contributions depending on the target modality (Bernier and Grafton, 2010), task demands (Badde et al., 2014, 2015) and sensory experience (Reuschel et al., 2012).

In depth, we found a preponderant space-centered representation that is commonly considered a more stable encoding mechanism (Medendorp and Crawford, 2002). In this case, the reach error patterns were more driven by the two eye/target configurations that shared the same spatial target position independently from the eye position. These two eye/target configurations presented the same reaching geometry because, in both cases, the participants reached peripheral targets that can be foveated or not, but, in general, the same trajectory dynamics were used (see Figure 6, first and last panels). This could indicate that the encoding of target depth is more influenced by the organization of trajectories and must rely on constant coordinates of the space that does not change with eye movements for example.

Many studies investigated coordinate systems used to encode the targets for memory-guided movements (Diedrichsen et al., 2004; Obhi and Goodale, 2005; Byrne and Crawford, 2010; Fielher et al., 2011). For example, Fielher et al. (2011) found that reach targets were updated relative to the position of the eyes when the movements were executed after different delays when no other external cue was available. However, other studies suggested the use of allocentric coordinate systems when movements were delayed (Diedrichsen et al., 2004; Obhi and Goodale, 2005). In particular, the presence of landmarks serves to improve the stability of the estimation of target position especially when the target of reaching is memorized (Obhi and Goodale, 2005). Furthermore, more recent work suggests that egocentric and allocentric information are integrated for memory-guided reaching movements by weighting each single input with respect to their reliability (Byrne and Crawford, 2010). In line with all these evidences, in our memory-guided reaching task, we found both space-centered and eye-centered representations differently distributed along the depth and direction dimensions.

By analyzing the kinematics of reaching configurations (see Figures 6, 7), we found that movement variability was different in depth and direction and in different eye/target configurations. We used the movement variability as a measure of trajectory strategies used by the participants across trial repetitions. Typically, this analysis has been applied to study the role of visual information on the reaching execution (Khan et al., 2002, 2003); here we have used it to identify common or different motor strategies for reaching in different eye/target configurations. In direction, we found that the variability distributions in eye-centered coordinates were completely different in the two configurations for the total duration of movement whereas in space-centered coordinates the variability distributions were similar. In depth, the variability distribution in eye-centered coordinates was similar for the first half of the movement (until the peak velocity) while in space-centered coordinate it was comparable for the entire movement. We found trajectory modifications also in the first part of movement: as several studies suggested that online processes do not influence movement at least to peak velocity (Elliott et al., 1999; Krakauer et al., 2000; Proteau and Isabelle, 2002), we can attribute these modifications to offline control processes (correction across trials) that are applied on subsequent trials (Khan et al., 2003).

All the differences in movement variability support two possible views. First, different strategies of movement are used in depth and direction when multiple eye/target configurations are used. This is evident by the distribution of variability for Constant gaze and Constant reach configurations, that was dramatically different in direction but partially similar in depth. Second, we cannot exclude the possibility that the differences between the two configurations were due to different shoulder/elbow postures during the movement. However, in general, a different mechanism in the organization of movement is evident in depth and direction. Previous behavioral studies showed that the encoding of depth and direction does not rely on shared networks in the brain during the execution of movement, but it is processed separately (Flanders et al., 1992; Gordon et al., 1994; Sainburg et al., 2003; Vindras et al., 2005; Bagesteiro et al., 2006; Van Pelt and Medendorp, 2008). However, other evidence suggested that movement amplitude (or depth) is processed later than the direction information during reaching preparation (Fu et al., 1995; Messier and Kalaska, 2000; Hadjidimitrakis et al., 2014a; Davare et al., 2015). The different findings identified here in depth and direction at two levels of behavioral investigation (reach errors and movement variability) are in agreement with both of these views. In particular, the majority of participants changed coordinate system from mixed in direction to space-centered in depth. As the mixed encoding represents not a final stage of coordinate transformations, but is important to support non linear computations required by these (Pouget and Snyder, 2000; Blohm, 2012), our data support the idea of a later processing of depth information with respect to the direction.

The differences found in the variability distribution and the consistency of these results across participants also suggest that, despite the presence of the same sensory and motor information in our reaching task, the eye/target configurations and the space dimensions strongly influence the control of movement. That indicates common trajectory strategies that go beyond the individual reaching behavior.

Studies of coordinate systems based on hand and target position demonstrated a correlation between the movement control and the coordinate system used. They have described that the brain extracts estimates of target and hand positions from a visual scene, calculates a difference vector between them, and uses this signal to compute the required motor commands (Cisek et al., 1998). Given that online motor corrections respond to changing visual information at time lags in the range of 100–120 ms (Day and Lyon, 2000; Franklin and Wolpert, 2008; Reichenbach et al., 2014), these computations have to be executed very quickly. In the present study, we can argue that to compensate the differences in movement variability, a mixed encoding was suitable to allow for fast non linear computations that are required for direction processing in order to quickly update the spatial coordinates of the upcoming movement. In depth, we found a space-centered representation and a more homogeneous distribution of variability along movement execution in eye-centered and space-centered coordinates. What we found may suggest that the reaching plans did not require modifications across eye/target configurations and a space-centered coordinate system provided a more stable encoding mechanism (Medendorp and Crawford, 2002). However, future studies must be addressed to clarify these aspects and to study directly the relationship between coordinate system and motor control.

### Comparison with Other Studies of Reaching in Depth

In several studies, it was found that participants did not use a stable non-retinal spatial mechanism to guide the arm movement but an eye-centered spatial mechanism, which is updated across eye movements for near and far space (Bock, 1986; Enright, 1995; Henriques et al., 1998; Lewald and Ehrenstein, 2000; Medendorp and Crawford, 2002). Medendorp and Crawford (2002) investigated only reaching towards targets located on a central straight line with respect to participants’ body and at eye level. The investigation of reaching target representation located at different depths and directions with respect to the body’s midline was introduced by Van Pelt and Medendorp (2008). They found that depth and direction are coded in a pure eye-centered coordinate system. In this study, the targets were presented in a horizontal plane at eye level, hence the reaching movements were from bottom to top (anti-gravity movements). In the present study, we found results that differ from those described above. These differences might originate from the different task conditions. In fact, we demonstrated that reach errors in direction mainly followed both eye-centered and space-centered representations and reach errors in depth were mainly characterized by representation shifted towards space-centered encoding. In our experiment, reaching movements were parallel to the touchscreen placed horizontally on the desk and the reaching movements were tested for targets located in the lower part of working space. This region of space is where most of the primate motor behavior takes place (Previc, 1998). Our setup was similar to natural conditions but more complex because the encoding of reaching targets not only requires an update of vergence signal but also an integration of vergence, elevation signals of the eyes and egocentric distance representation of the target. The discrepancy between the present work and the previous one may be caused by all these reasons.

## Conclusion

This study shows that when eye and hand are dissociated in depth and direction, the behavioral encoding of target positions is based on both eye-centered and space-centered representations. Interestingly, when we consider changes along depth dimension, the influence of space-centered representation becomes higher than the influence of changes in direction. This different balance of space encoding mechanisms represents a suitable method used by the brain to adapt to the possible perturbations that can occur during the movement and provides the motor system with necessary information to accurately correct the movement. The variability distribution along the movement execution was influenced by the eye/target configurations as well as by depth and direction suggesting that participants adopted different strategies according to movement geometries and task demands. Finally, our behavioral results support the hypothesis that the brain needs the conjunct contribution of multiple coordinate systems to efficiently compensate the variety of corrections required by the complex metrics of reaching movements executed to targets located at different depths and directions.

## Author Contributions

PF and AB designed the research; wrote the article. AB and VP recorded the data; analyzed the data.

## Conflict of Interest Statement

The authors declare that the research was conducted in the absence of any commercial or financial relationships that could be construed as a potential conflict of interest.

## References

[B2] Andersen R. A. R. Buneo C. A. C. (2002). Intentional maps in posterior parietal cortex. Annu. Rev. Neurosci. 25, 189–220. 10.1146/annurev.neuro.25.112701.142922 12052908

[B1] Andersen R. A. Snyder L. H. Li C. S. Stricanne B. (1993). Coordinate transformations in the representation of spatial information. Curr. Opin. Neurobiol. 3, 171–176. 10.1016/0959-4388(93)90206-e 8513228

[B3] Avillac M. Denève S. Olivier E. Pouget A. Duhamel J.-R. (2005). Reference frames for representing visual and tactile locations in parietal cortex. Nat. Neurosci. 8, 941–949. 10.1038/nn1480 15951810

[B4] Badde S. Heed T. Röder B. (2014). Processing load impairs coordinate integration for the localization of touch. Atten. Percept. Psychophys. 76, 1136–1150. 10.3758/s13414-013-0590-2 24550040

[B5] Badde S. Röder B. Heed T. (2015). Flexibly weighted integration of tactile reference frames. Neuropsychologia 70, 367–374. 10.1016/j.neuropsychologia.2014.10.001 25447059

[B6] Bagesteiro L. B. Sarlegna F. R. Sainburg R. L. (2006). Differential influence of vision and proprioception on control of movement distance. Exp. Brain Res. 171, 358–370. 10.1007/s00221-005-0272-y 16307242 PMC10710692

[B7] Batista A. P. Buneo C. A. Snyder L. H. Andersen R. A. (1999). Reach plans in eye-centered coordinates. Science 285, 257–260. 10.1126/science.285.5425.257 10398603

[B8] Battaglia-Mayer A. Caminiti R. Lacquaniti F. Zago M. (2003). Multiple levels of representation of reaching in the parieto-frontal network. Cereb. Cortex 13, 1009–1022. 10.1093/cercor/13.10.1009 12967918

[B9] Bernier P. M. Grafton S. T. (2010). Human posterior parietal cortex flexibly determines reference frames for reaching based on sensory context. Neuron 68, 776–788. 10.1016/j.neuron.2010.11.002 21092865

[B10] Beurze S. M. Toni I. Pisella L. Medendorp W. P. (2010). Reference frames for reach planning in human parietofrontal cortex. J. Neurophysiol. 104, 1736–1745. 10.1152/jn.01044.2009 20660416

[B11] Beurze S. M. Van Pelt S. Medendorp W. P. (2006). Behavioral reference frames for planning human reaching movements. J. Neurophysiol. 96, 352–362. 10.1152/jn.01362.2005 16571731

[B12] Blohm G. (2012). Simulating the cortical 3D visuomotor transformation of reach depth. PLoS One 7:e41241. 10.1371/journal.pone.0041241 22815979 PMC3397995

[B13] Bock O. (1986). Contribution of retinal versus extraretinal signals towards visual localization in goal-directed movements. Exp. Brain Res. 64, 476–482. 10.1007/bf00340484 3803485

[B15] Bosco A. Breveglieri R. Hadjidimitrakis K. Galletti C. Fattori P. (2016). Reference frames for reaching when decoupling eye and target position in depth and direction. Sci. Rep. 6:21646. 10.1038/srep21646 26876496 PMC4753502

[B16] Bosco A. Breveglieri R. Reser D. Galletti C. Fattori P. (2015a). Multiple representation of reaching space in the medial posterior parietal area V6A. Cereb. Cortex 25, 1654–1667. 10.1093/cercor/bht420 24421176

[B17] Bosco A. Lappe M. Fattori P. (2015b). Adaptation of saccades and perceived size after trans-saccadic changes of object size. J. Neurosci. 35, 14448–14456. 10.1523/JNEUROSCI.0129-15.2015 26511237 PMC6605459

[B18] Brainard D. H. (1997). The psychophysics toolbox. Spat. Vis. 10, 433–436. 10.1163/156856897x00357 9176952

[B19] Bremner L. R. Andersen R. A. (2014). Temporal analysis of reference frames in parietal cortex area 5d during reach planning. J. Neurosci. 34, 5273–5284. 10.1523/JNEUROSCI.2068-13.2014 24719105 PMC3983803

[B20] Buchholz V. N. Jensen O. Medendorp W. P. (2013). Parietal oscillations code nonvisual reach targets relative to gaze and body. J. Neurosci. 33, 3492–3499. 10.1523/JNEUROSCI.3208-12.2013 23426676 PMC6619549

[B22] Buneo C. A. Batista A. P. Jarvis M. R. Andersen R. A. (2008). Time-invariant reference frames for parietal reach activity. Exp. Brain Res. 188, 77–89. 10.1007/s00221-008-1340-x 18368398

[B21] Buneo C. A. Jarvis M. R. Batista A. P. Andersen R. A. (2002). Direct visuomotor transformations for reaching. Nature 416, 632–636. 10.1038/416632a 11948351

[B23] Byrne P. Crawford J. (2010). Cue reliability and a landmark stability heuristic determine relative weighting between egocentric and allocentric visual information in memory-guided reach. J. Neurophysiol. 103, 3054–3069. 10.1152/jn.01008.2009 20457858

[B24] Carey D. P. Hargreaves E. L. Goodale M. A. (1996). Reaching to ipsilateral or contralateral targets: within-hemisphere visuomotor processing cannot explain hemispatial differences in motor control. Exp. Brain Res. 112, 496–504. 10.1007/bf00227955 9007551

[B25] Carlton L. G. (1992). “Visual processing time and the control of movement,” in Vision and Motor Control, eds Proteau L. Elliott D. (Amsterdam: Elsevier), 3–31.

[B26] Chang S. W. C. Snyder L. H. (2010). Idiosyncratic and systematic aspects of spatial representations in the macaque parietal cortex. Proc. Natl. Acad. Sci. U S A 107, 7951–7956. 10.1073/pnas.0913209107 20375282 PMC2867917

[B27] Cisek P. Grossberg S. Bullock D. (1998). A cortico-spinal model of reaching and proprioception under multiple task constraints. J. Cogn. Neurosci. 10, 425–444. 10.1162/089892998562852 9712674

[B28] Cohen Y. E. Andersen R. A. (2002). A common reference frame for movement plans in the posterior parietal cortex. Nat. Rev. Neurosci. 3, 553–562. 10.1038/nrn873 12094211

[B29] Davare M. Zénon A. Desmurget M. Olivier E. (2015). Dissociable contribution of the parietal and frontal cortex to coding movement direction and amplitude. Front. Hum. Neurosci. 9:241. 10.3389/fnhum.2015.00241 25999837 PMC4422032

[B30] Day B. L. Lyon I. N. (2000). Voluntary modification of automatic arm movements evoked by motion of a visual target. Exp. Brain Res. 130, 159–168. 10.1007/s002219900218 10672469

[B31] Deneve S. Latham P. E. Pouget A. (2001). Efficient computation and cue integration with noisy population codes. Nat. Neurosci. 4, 826–831. 10.1038/90541 11477429

[B32] Dessing J. C. Crawford J. D. Medendorp W. P. (2011). Spatial updating across saccades during manual interception. J. Vis. 11:4. 10.1167/11.10.4 21900372

[B33] Diedrichsen J. Nambisan R. Kennerley S. Ivry R. (2004). Independent on-line control of the two hands during bimanual reaching. Eur. J. Neurosci. 19, 1643–1652. 10.1111/j.1460-9568.2004.03242.x 15066160

[B34] Elliott D. Binsted G. Heath M. (1999). The control of goal-directed limb movements: correcting errors in the trajectory. Hum. Mov. Sci. 18, 121–136. 10.1016/s0167-9457(99)00004-4

[B35] Enright J. T. (1995). The non-visual impact of eye orientation on eye-hand coordination. Vision Res. 35, 1611–1618. 10.1016/0042-6989(94)00260-s 7667918

[B36] Fielher K. Schütz I. Henriques D. (2011). Gaze-centered spatial updating of reach targets across different memory delays. Vision Res. 51, 890–897. 10.1016/j.visres.2010.12.015 21219923

[B37] Flanders M. Tillery S. I. H. Soechting J. F. (1992). Early stages in a sensorimotor transformation. Behav. Brain Sci. 15, 309–320. 10.1017/s0140525x00068813

[B38] Franklin D. W. Wolpert D. M. (2008). Specificity of reflex adaptation for task-relevant variability. J. Neurosci. 28, 14165–14175. 10.1523/JNEUROSCI.4406-08.2008 19109499 PMC2636902

[B39] Fu Q. G. Flament D. Coltz J. D. Ebner T. J. (1995). Temporal encoding of movement kinematics in the discharge of primate primary motor and premotor neurons. J. Neurophysiol. 73, 836–854. 7760138 10.1152/jn.1995.73.2.836

[B40] Gentilucci M. Benuzzi F. Gangitano M. Grimaldi S. (2001). Grasp with hand mouth: a kinematic study on healthy subjects. J. Neurophysiol. 86, 1685–1699. 11600632 10.1152/jn.2001.86.4.1685

[B41] Gordon J. Ghilardi M. F. Cooper S. E. Ghez C. (1994). Accuracy of planar reaching movements—II. Systematic extent errors resulting from inertial anisotropy. Exp. Brain Res. 99, 112–130. 10.1007/bf00241416 7925785

[B42] Hadjidimitrakis K. Bertozzi F. Breveglieri R. Bosco A. Galletti C. Fattori P. (2014a). Common neural substrate for processing depth and direction signals for reaching in the monkey medial posterior parietal cortex. Cereb. Cortex 24, 1645–1657. 10.1093/cercor/bht021 23382514

[B43] Hadjidimitrakis K. Bertozzi F. Breveglieri R. Fattori P. Galletti C. (2014b). Body-centered, mixed, but not hand-centered coding of visual targets in the medial posterior parietal cortex during reaches in 3D space. Cereb. Cortex 24, 3209–3220. 10.1093/cercor/bht181 23853212

[B44] Henriques D. Y. Klier E. M. Smith M. A. Lowy D. Crawford J. D. (1998). Gaze-centered remapping of remembered visual space in an open-loop pointing task. J. Neurosci. 18, 1583–1594. 9454863 10.1523/JNEUROSCI.18-04-01583.1998 PMC6792733

[B45] Kasuga S. Telgen S. Ushiba J. Nozaki D. Diedrichsen J. (2015). Learning feedback and feedforward control in a mirror-reversed visual environment. J. Neurophysiol. 114, 2187–2193. 10.1152/jn.00096.2015 26245313 PMC4600966

[B46] Khan M. A. Elliott D. Coull J. Chua R. Lyons J. (2002). Optimal control strategies under different feedback schedules: kinematic evidence. J. Mot. Behav. 34, 45–57. 10.1080/00222890209601930 11880249

[B47] Khan M. A. Lawrence G. Fourkas A. Franks I. M. Elliott D. Pembroke S. (2003). Online versus offline processing of visual feedback in the control of movement amplitude. Acta Psychol. (Amst) 113, 83–97. 10.1016/s0001-6918(02)00156-7 12679045

[B48] Krakauer J. W. Pine Z. M. Ghilardi M. F. Ghez C. (2000). Learning of visuomotor transformations for vectorial planning of reaching trajectories. J. Neurosci. 20, 8916–8924. 11102502 10.1523/JNEUROSCI.20-23-08916.2000 PMC6773094

[B49] Lewald J. Ehrenstein W. H. (2000). Visual and proprioceptive shifts in perceived egocentric direction induced by eye-position. Vision Res. 40, 539–547. 10.1016/s0042-6989(99)00197-2 10820612

[B50] Marzocchi N. Breveglieri R. Galletti C. Fattori P. (2008). Reaching activity in parietal area V6A of macaque: eye influence on arm activity or retinocentric coding of reaching movements? Eur. J. Neurosci. 27, 775–789. 10.1111/j.1460-9568.2008.06021.x 18279330 PMC2268963

[B51] McGuire L. M. M. Sabes P. N. (2009). Sensory transformations and the use of multiple reference frames for reach planning. Nat. Neurosci. 12, 1056–1061. 10.1038/nn.2357 19597495 PMC2749235

[B52] McGuire L. M. Sabes P. N. (2011). Heterogeneous representations in the superior parietal lobule are common across reaches to visual and proprioceptive targets. J. Neurosci. 31, 6661–6673. 10.1523/JNEUROSCI.2921-10.2011 21543595 PMC3100795

[B53] Medendorp W. P. Crawford J. D. (2002). Visuospatial updating of reaching targets in near and far space. Neuroreport 13, 633–636. 10.1097/00001756-200204160-00019 11973460

[B54] Messier J. Kalaska J. F. (2000). Covariation of primate dorsal premotor cell activity with direction and amplitude during a memorized reaching task. J. Neurophysiol. 84, 152–165. 10899193 10.1152/jn.2000.84.1.152

[B55] Mueller S. Fiehler K. (2016). Mixed body- and gaze-centered coding of proprioceptive reach targets after effector movement. Neuropsychologia 87, 63–73. 10.1016/j.neuropsychologia.2016.04.033 27157885

[B56] Mullette-Gillman O. A. Cohen Y. E. Groh J. M. (2005). Eye-centered, head-centered and complex coding of visual and auditory targets in the intraparietal sulcus. J. Neurophysiol. 94, 2331–2352. 10.1152/jn.00021.2005 15843485

[B57] Mullette-Gillman O. A. Cohen Y. E. Groh J. M. (2009). Motor-related signals in the intraparietal cortex encode locations in a hybrid, rather than eye-centered reference frame. Cereb. Cortex 19, 1761–1775. 10.1093/cercor/bhn207 19068491 PMC2705694

[B59] Obhi S. Goodale M. (2005). The effects of landmarks on the performance of delayed and real-time pointing movements. Exp. Brain Res. 167, 335–344. 10.1007/s00221-005-0055-5 16041512

[B60] Oldfield R. C. (1971). The assessment and analysis of handedness: the Edinburgh inventory. Neuropsychologia 9, 97–113. 10.1016/0028-3932(71)90067-4 5146491

[B61] Pelisson D. Prablanc C. Goodale M. Jeannerod M. (1986). Visual control of reaching movements without vision of the limb. Exp. Brain Res. 62, 303–311. 10.1007/bf00238848 3709715

[B62] Peña J. L. Konishi M. (2001). Auditory spatial receptive fields created by multiplication. Science 292, 249–252. 10.1126/science.1059201 11303092

[B63] Pesaran B. Nelson M. J. Andersen R. A. (2006). Dorsal premotor neurons encode the relative position of the hand, eye, and goal during reach planning. Neuron 51, 125–134. 10.1016/j.neuron.2006.05.025 16815337 PMC3066049

[B64] Pesaran B. Nelson M. J. Andersen R. A. (2010). A relative position code for saccades in dorsal premotor cortex. J. Neurosci. 30, 6527–6537. 10.1523/JNEUROSCI.1625-09.2010 20463216 PMC2887302

[B65] Piserchia V. Breveglieri R. Hadjidimitrakis K. Bertozzi F. Galletti C. Fattori P. (2017). Mixed body/hand reference frame for reaching in 3D space in macaque parietal area PEc. Cereb. Cortex 27, 1976–1990. 10.1093/cercor/bhw039 26941385

[B66] Pouget A. Snyder L. H. (2000). Computational approaches to sensorimotor transformations. Nat. Neurosci. 3, 1192–1198. 10.1038/81469 11127837

[B67] Previc F. H. (1998). The neuropsychology of 3-D space. Psychol. Bull. 124, 123–164. 10.1037/0033-2909.124.2.123 9747184

[B68] Proteau L. Isabelle G. (2002). On the role of visual afferent information for the control of aiming movements towards targets of different sizes. J. Mot. Behav. 34, 367–384. 10.1080/00222890209601954 12446251

[B69] Reichenbach A. Franklin D. W. Zatka-Haas P. Diedrichsen J. (2014). A dedicated binding mechanism for the visual control of movement. Curr. Biol. 24, 780–785. 10.1016/j.cub.2014.02.030 24631246 PMC3988841

[B70] Reuschel J. Rösler F. Henriques D. Fiehler K. (2012). Spatial updating depends on gaze direction even after loss of vision. J. Neurosci. 32, 2422–2429. 10.1523/JNEUROSCI.2714-11.2012 22396416 PMC6621798

[B71] Roy A. C. Paulignan Y. Farnè A. Jouffrais C. Boussaoud D. (2000). Hand kinematics during reaching and grasping in the macaque monkey. Behav. Brain Res. 117, 75–82. 10.1016/s0166-4328(00)00284-9 11099760

[B72] Sainburg R. L. Lateiner J. E. Latash M. L. Bagesteiro L. B. (2003). Effects of altering initial position on movement direction and extent. J. Neurophysiol. 89, 401–415. 10.1152/jn.00243.2002 12522189 PMC10709819

[B73] Scherberger H. Goodale M. A. Andersen R. A. (2003). Target selection for reaching and saccades share a similar behavioral reference frame in the macaque. J. Neurophysiol. 89, 1456–1466. 10.1152/jn.00883.2002 12612028

[B74] Snyder L. Batista A. Andersen R. (1997). Coding of intention in the posterior parietal cortex. Nature 386, 167–170. 10.1038/386167a0 9062187

[B75] Soechting J. F. Flanders M. (1992). Moving in three-dimensional space: frames of reference, vectors, and coordinate systems. Annu. Rev. Neurosci. 15, 167–191. 10.1146/annurev.neuro.15.1.167 1575441

[B76] Stricanne B. Andersen R. A. Mazzoni P. (1996). Eye-centered, head-centered, and intermediate coding of remembered sound locations in area LIP. J. Neurophysiol. 76, 2071–2076. 8890315 10.1152/jn.1996.76.3.2071

[B77] Tramper J. J. Medendorp W. P. (2015). Parallel updating and weighting of multiple spatial maps for visual stability during whole-body motion. J. Neurophysiol. 114, 3211–3219. 10.1152/jn.00576.2015 26490289 PMC4686301

[B78] Van Pelt S. Medendorp W. P. (2008). Updating target distance across eye movements in depth. J. Neurophysiol. 99, 2281–2290. 10.1152/jn.01281.2007 18353912

[B79] Vindras P. Desmurget M. Viviani P. (2005). Error parsing in visuomotor pointing reveals independent processing of amplitude and direction. J. Neurophysiol. 94, 1212–1224. 10.1152/jn.01295.2004 15857965

